# Traumatic unilateral lumbosacral jumped facet without fracture in a child – presentation of a safe treatment strategy for a rare injury

**DOI:** 10.1186/1754-9493-2-29

**Published:** 2008-11-10

**Authors:** Oszkar Szentirmai, Joshua Seinfeld, Kathryn Beauchamp, Vikas Patel

**Affiliations:** 1 Department of Neurosurgery, Denver Health Medical Center, University of Colorado at Denver and Health Sciences Center, Denver, Colorado, USA; 2 Department of Orthopaedics, Denver Health Medical Center, University of Colorado at Denver and Health Sciences Center, Denver, Colorado, USA

## Abstract

The vast majority of pediatric lumbosacral spondylolisthesis have developmental etiology. Of the very rare type of pediatric lumbosacral facet dislocations, there are only three reported cases of a pediatric unilateral jumped facet injury. All of these cases are associated with fracture dislocation of L5-S1. Hyperflexion with rotation is thought to provoke this uncommon type of spine injury.

The authors report the first pediatric patient reported in literature to date with a traumatic unilateral jumped facet at the lumbosacral joint without fracture. The presentation, surgical treatment, hospital course, outcome and management options with the review of the literature is summarized.

## Background

Unilateral jumped (or locked) facet injuries are consequences of massive forces with a rotational component in the setting of hyperflexion. Most patients suffer minor or no neurologic injury. Unilateral lumbosacral facet dislocation injuries are infrequent and are often associated with fractures at L5-S1 and other segments of the spinal column. We found a total of approximately 50 traumatic lumbosacral facet dislocation cases reported in the literature. 21 cases are unilateral lumbosacral injuries. 3 of these cases are pediatric and are described as unilateral fracture-dislocations [1-5]. To our knowledge, this is the first case reported in the PubMed literature database with an L5-S1 unilateral jumped facet injury with anterolisthesis but without associated L5-S1 fracture.

Sporadic early case reports initially suggested conservative management with cast immobilization as a viable treatment option. More recent reports advocate anterior and/or posterior surgical reduction with instrumentation. Here we report our own experience with stand alone open posterior reduction and instrumentation with iliac crest bone grafting that resulted in excellent clinical outcome.

## Case report

**Figure 1:**
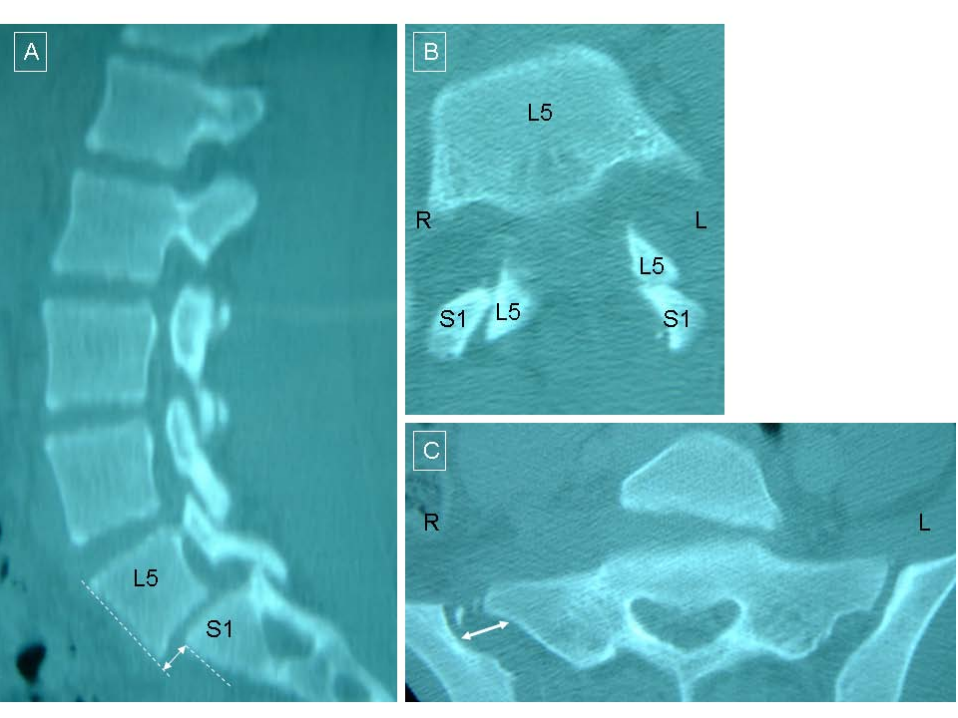
**Pre-operative images**. Panel a) Sagittal CT reconstruction revealed the anterolisthesis of L5 on S1. Axial CT at L5 in Panel b) shows left L5-S1 jumped facet. Panel c) shows the right > left sacroiliac joint dislocation.

A 14-year-old girl was an unrestrained driver in a roll-over accident. She ejected from the vehicle without loss of consciousness. On arrival to the hospital she was found to be neurologically intact but with limited range of motion in both legs due to pain in her back and pelvis. Her workup revealed an L5-S1 anterolisthesis (Figure 1/A), a left L5-S1 unilateral jumped facet without evidence of fracture (Figure 1/B), a right greater than left sacroiliac dislocation (Figure 1/C), a left iliac wing fracture, multiple lumbar transverse process fractures, a C7 transverse process fracture and a T1 compression fracture. As seen in Figure 1, there was anterolisthesis between L5-S1 without evidence of pars interarticularis fracture. She was taken to the operating room on the day after her injury for posterior reduction and instrumentation. Extensive soft tissue injury was noted with rupture of the lumbar fascia, facet capsule and interspinous ligaments.

**Figure 2:**
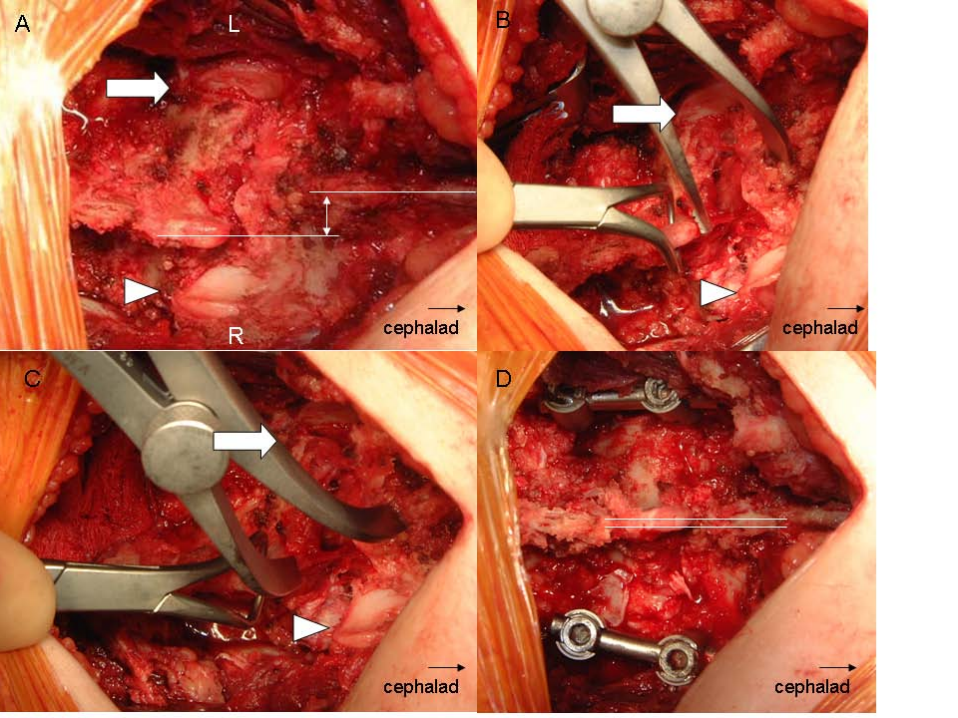
**Intra-operative images**. Panel a) Left L5-S1 jumped facet and rotational anterolisthesis (lines over the spinous processes). Panel b) shows partial reduction during distraction. Panel c) Reduction of the left L5/S1 joint that was fixed into position with posterior instrumentation as shown in Panel d) with re-alignment of the axis (double lines).

**Figure 3:**
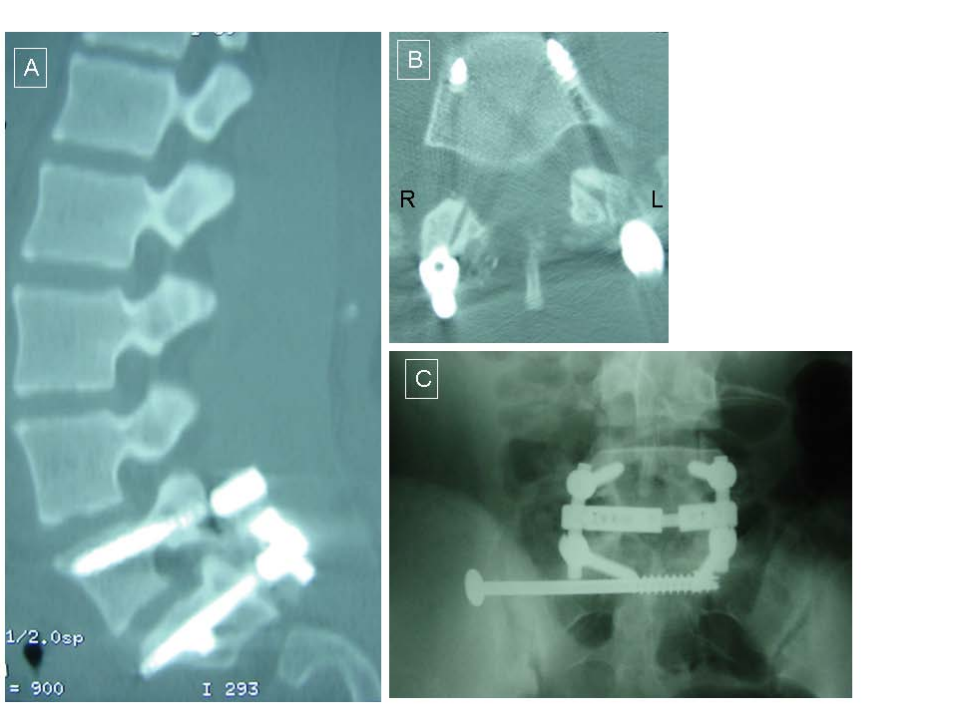
**Post-operative images**. Panel a) Sagittal CT reconstruction following reduction of the anterolisthesis of L5 on S1 with posterior instrumentation. Axial CT scan in Panel b) shows the pedicular screws of L5 with anatomical reduction of the left locked L5-S1 facet joint. In Panel c), AP X-ray of the lumbosacral area with reduced right sacroiliac joint following SI joint screw fixation.

The left unilateral L5-S1 jumped facet injury was confirmed intra-operatively without signs of pars defects (Figure 2/A). A large left paraspinous hematoma was evacuated that resulted from muscular tear at the time of injury. Reduction was unsuccessfully attempted using a combination of laminar spreader distraction between the L5 and S1 spinous processes and a towel clamp on L5 for posterior traction (Figure 2/B). Even though the facets could be unlocked, reduction was not possible. A spondylolisthesis reduction device (Aesculap Inc, Center Valley, PA) was used on the left side to reduce the rotational deformity as the vertebrae were distracted, reduced, and then compressed. Once the facet dislocation was atraumatically reduced and well approximated (Figure 2/C), 6.0 mm pedicle screws and rods (Aesculap Spine) were used for posterior instrumentation of L5-S1 (Figure 2/D). Autologous bone graft was harvested from the iliac crest and was applied bilaterally for optimal fusion. Post-operative imaging revealed proper pedicle screw placement and realignment of the right L5-S1 facet joint (Figure 3/A and 3/B). Two days following her accident, she was taken back to the operating room for right percutaneous sacroiliac screw placement for the reduction of the SI joint dislocation (Figure 3/C). The same week she was discharged home neurologically intact but with some subjective left leg weakness and numbness below the knee. She used a lumbar corset while ambulating for 6 weeks following surgery. On her 2 year follow-up, the patient was asymptomatic, pain free, and was able to return to her prior active lifestyle which includes school and sports. She had a small patch of persistent numbness on the left shin which did not impede her activities.

## Discussion

Traumatic unilateral facet dislocations decrease in frequency towards the caudal spinal column. Watson-Jones proposed hyperextension as the underlying cause of lumbo-sacral fracture-dislocations in the 1940s[6]. Dewey and Browne further explained that spondylolisthesis is secondary to anterior vector forces[7] while Samberg provided the most compelling explanation of rotational forces with hyperflexion[8] as the major underlying mechanism. With only a handful of similar cases published in the literature, it is only proper to correlate the conclusion drawn from sporadic clinical experiences with the results of controlled experiments in animal and cadaver models on the forces involved to elicit these types of spinal column injuries. There are only twelve published case review papers on unilateral lumbosacral fracture-dislocations (21 total cases). Thoracolumbar and cervical unilateral facet dislocations are more common [9-12]. Pediatric sacroiliac unilateral jumped facet injuries are rare with only three published cases to date, all involving fracture at the level of the jumped facet. Management recommendations in this age group are not well established. Isolated ligamentous injury associated with jumped facet and without fracture, as presented in this manuscript, has not been published in the pediatric literature. Instability of all three vertebral columns necessitates surgical reduction with instrumentation. There are numerous case reports and review series on bilateral facet dislocations with recommendations for CT scanning of the spine, early reduction with lumbosacral arthrodesis and posterolateral bone grafting with instrumentation[4,13,14]. In bilateral facet injuries careful assessment for possible intervertebral disc herniation leading to cauda equina syndrome is warranted with MRI imaging and possible canal exploration[15]. The high energy forces leading to a lumbosacral unilateral spondylolisthesis is likely to cause other injuries. In our patient we found a large paraspinous hematoma, multiple lumbar transverse process fractures, an iliac wing fracture and a T1 compression fracture. Many of these associated injuries can go undetected without a thorough workup. Less than one third of the patients in the reported lumbosacral dislocation cases presented with impaired neurologic function, suggesting that nerve root evulsion or severe canal compromise is infrequent in this type of spine injury. Our case suggests that there might be a higher incidence of associated injuries than previously published in similar traumatic pathologies of the lumbosacral spine.

Although some authors have advocated anterior or anterior-posterior 360 degree spinal fusion with instrumentation[14,16-18], in this case we achieved good results with only posterior-lateral fusion with instrumentation. This was deemed appropriate at the time of surgery based on a stable reduction with near anatomic re-approximation of the left L5-S1 jumped facet. The integrity of the L5-S1 disc was considered, but thought to provide adequate anterior column support in combination with satisfactory pedicle screw placement. Long term follow up will be important as this patient ages to assess the natural history of the disc and possible sequelae of not removing this at the time of surgery.

The rotational deformity was quite significant (approximately 30 degrees) and thus the patient was not expected to do well with only cast or brace treatment. The difficulty of surgical reduction also implied that closed reduction, though not attempted, would not have been successful. The difficulty of maintaining the achieved reduction of the L5-S1 jumped facet intra-operatively argues for the inclusion of a reduction device or spondylolisthesis reduction screws in the pre-operative surgical plan.

In summary, based on the available literature and our experience, stand alone posterior instrumentation with arthrodesis can provide excellent clinical outcome following unilateral spondylolisthesis in a pediatric patient.

## Conclusion

Stand alone posterior instrumentation with arthrodesis is a safe treatment in a pediatric patient. This approach avoids the inherent risks associated with anterior exposure of the lumbosacral junction.

## Competing interests

The authors declare that they have no competing interests.

## Authors' contributions

OS, JS and VP made substantial contributions to conception and design of this paper. KB revised the manuscript and formatted for publication.
